# Salt Distribution in Raw Sheep Milk Cheese during Ripening and the Effect on Proteolysis and Lipolysis

**DOI:** 10.3390/foods8030100

**Published:** 2019-03-17

**Authors:** Olaia Estrada, Agustín Ariño, Teresa Juan

**Affiliations:** 1 Centro de Investigación y Tecnología Agroalimentaria de Aragón (CITA), 50059 Zaragoza, Spain; oestrada@cita-aragon.es (O.E.); tjuan@cita-aragon.es (T.J.); 2 Instituto Agroalimentario de Aragón-IA2 (Universidad de Zaragoza-CITA), Facultad de Veterinaria, 50013 Zaragoza, Spain

**Keywords:** raw sheep milk cheese, artisanal cheese, salt distribution, proteolysis, lipolysis, ripening, sampling zone

## Abstract

The salt distribution process in artisanal sheep cheese with an innovative shape of eight lobes was investigated. The cheese was subjected to two brining conditions: 24 h with brine at 16°Baumé and 12 h at 22°Baumé. The chemical composition (pH, water activity, dry matter, fat, and protein content), proteolysis (nitrogen fractions and free amino acids), and lipolysis (free fatty acids) were evaluated in two sampling zones (internal and external) at 1, 15, 30, 60, 90, 120, 180, and 240 days of ripening. The whole cheese reached a homogeneous salt distribution at 180 days of ripening. Brining conditions did not have an influence on the rate of salt penetration, but on the final sodium chloride (NaCl) content. Cheese with higher salt content (3.0%) showed increased proteolysis and lipolysis as compared to cheese with lower salt content (2.2%). Proteolysis index and total free fatty acids did not differ significantly (*p* > 0.05) between internal and external zones of cheese. It is suggested that producers start marketing this artisanal cheese at 6 months of ripening, when it has uniform composition and salt distribution.

## 1. Introduction

The consumer interest in and demand for artisanal dairy products are increasing. The development of these products elaborated on a small scale is receiving more consideration from European policy-makers due to their important role in the local agricultural economy and their contribution to development and sustainability, especially in rural areas.

Cheeses from Teruel (Spain) have been acclaimed since time immemorial. Miguel de Cervantes mentioned Tronchon cheese from Teruel in one of the greatest works of literature, *The Ingenious Nobleman Sir Quixote of La Mancha*, in 1615. Teruel cheese is a hard cheese produced with raw sheep or goat milk (not mixed), maintaining the traditional manufacturing process but including an innovative mold of eight lobes, unique in the market. This new shape, joining tradition and innovation, was designed and patented by producers to attract the attention of consumers.

In artisanal cheese varieties, flavor is more intense and rich than in industrially processed cheeses due to the native microbiota that develop peculiar organoleptic characteristics, which are appreciated by consumers [1]. Salt content plays a key role in the complex biochemical reactions during cheese ripening due to its effect on microbial growth, enzyme activity, and syneresis [2]. The main methods of salting are adding sodium chloride to the curd (dry salting), rubbing the salt on the surface of the cheese (dry surface rubbing), or immersing the cheese in a brine solution of sodium chloride and water (brining). In Spain, most semi-hard and hard cheeses are salted by brining.

During brining and ripening, salt diffusion and outward migration of water take place until equilibrium is reached throughout the cheese. Although this is a crucial step in cheese manufacture, there may be considerable variability in brining conditions, such as time, salinity, and temperature, for the same cheese variety [3]. In spite of the effect of salt content on organoleptic characteristics of cheese, only five out of 28 Spanish cheeses with Protected Designation of Origin (PDO) status have established the sodium chloride content in their respective regulations. Regulations for PDO Manchego (<2.3%), PDO Torta del Casar (<3%), PDO Valdeon (<3.5%), and PDO L’Alt Urgell y La Cerdanya (1.5–2.5%) cheeses detail sodium chloride criteria.

Proteolysis and lipolysis are the most complex biochemical changes that occur during cheese ripening. A reduced salt content in cheese has been related to increased proteolysis and sensory defects such as reduction of firmness, excessive acidity, and bitter taste [4,5]. Lipolysis seems to have less correlation with the salt content of cheese; free fatty acids released during ripening were not modified by salt reduction in Cheddar cheese [6]. Only a few studies have investigated the effects of salt distribution on proteolysis and lipolysis during cheese ripening. The sampling zone (internal and external) has been considered in large-format cow’s milk cheeses such as Parmigiano-Reggiano (>30 kg) [7,8], Reggianito Argentino (5–10 kg weight) [9], and Port Salut Argentino [10]. However, there are no studies available on the effect of salt distribution on the phenomena of proteolysis and lipolysis during ripening in raw sheep milk cheese. Additionally, the shape of cheese may affect the rate of salt absorption [2], so it is important to elucidate details of how the salt is distributed in Teruel cheese, with its innovative multilobed shape.

The aim of the current study was to assess the salt distribution process in a hard cheese (~4 kg) made with raw sheep milk and ripened for 240 days, elaborated under artisanal cheesemaking methods and with a multilobed shape. Two brining conditions were tested (24 h at 16°Baumé versus 12 h at 22°Baumé). In addition, the effect of salt content on chemical composition, proteolysis, and lipolysis was evaluated at two sampling zones (internal and external). The results obtained are intended to provide producers with important scientific information on the optimal time to put the cheese on the market.

## 2. Materials and Methods

### 2.1. Cheese-Making Procedure

**Figure 1:**
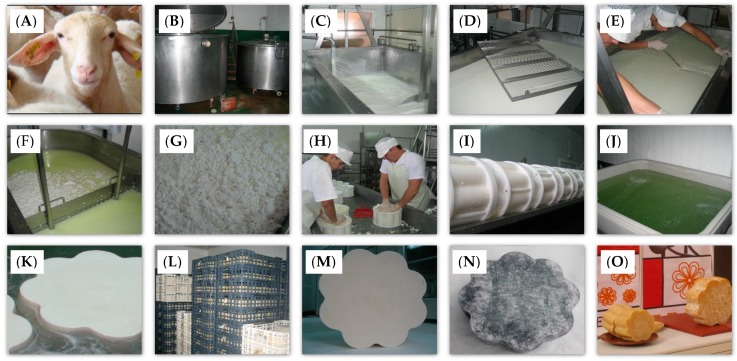
Cheesemaking procedure of artisanal raw sheep milk cheese: (**A**) Assaf sheep; (**B**) milk cooling tank; (**C**) cheese vat; (**D**) curd cutter; (**E**) manual cutting; (**F**) draining the whey; (**G**) dry curd; (**H**) manual molding; (**I**) pressing; (**J**) brine tank; (**K**) brining; (**L**) maturation chamber; (**M**) one-day ripened Teruel cheese; (**N**) three months of ripening; (**O**) six months of ripening.

In order to select the conditions for the study, we visited 10 artisanal dairies belonging to the Asociación Turolense de Productores de Leche y Queso (Association of Milk and Cheese Producers from Teruel). The cheesemaking process was analyzed and showed many similarities among dairies; most of the differences were due to the salting process. Thus, two dairies representing the two most common salting procedures were selected to carry out the experiments, in which the main objective was to study the degree of uniformity in the rate of absorption of salt by both brining conditions, considering the new shape with eight lobes. The operating conditions at both dairies were identical in relation to the type and amount of rennet used, the type and inoculation dosage of starter culture, the duration and temperature of coagulation, curd cutting and agitation, grain size of the curd, and molding and pressing. The only difference between dairies was the brining conditions, as described below. The cheeses were manufactured according to traditional methods (Figure 1) by two farmhouse cheese producers located in Teruel, Spain (named dairy plants D1 and D2), with raw sheep milk from Assaf breed produced by their own livestock. The chemical composition of the milk (g/100 g) was fat 6.79 ± 1.51, protein 5.30 ± 0.52, lactose 4.81 ± 0.66, and nonfat solids 11.03 ± 0.30. Milk was stored at 4 ± 1 °C until used (48 h maximum).

Lyophilized heterofermentative culture (CHOOZIT 4001/4002 MA, Danisco) was dispersed in approximately 300 mL of fresh milk and then added to the manufacturing milk (2%, w/v) at 20 °C. Starter cultures contained *Lactococcus lactis* subsp. *lactis*, *Lactococcus lactis* subsp. *cremoris*, *Lactococcus lactis* subsp. *lactis* biovar diacetylactis, and *Streptococcus thermophilus*. Milk was heated to 30 ± 1 °C and bovine rennet (~70% chymosin, ~30% pepsine; Arroyo, Spain) was added at a ratio of 25 mL/100 L to obtain clotting within 30–35 min. When the master cheesemaker observed the proper consistency of the curd, the coagulum was cut into rice/chickpea-sized grains (5–10 mm) and stirred for 45 min at 35 °C. Then, the whey was drained off and the curd was manually placed in microperforated plastic molds with an 8-lobed shape. Cheeses were pressed in a horizontal pneumatic press increasing from 2 to 3.5 kPa for nearly 6 h and then salted by immersion in brine (NaCl solution) at 10 °C. At dairy D1, the brine strength was 16°Baumé for 24 h, and at D2 the brine strength was 22°Baumé for 12 h. After 2–3 days in the airing area, the cheeses were stored in a maturation chamber at 10–12 °C and 80–85% relative humidity and were turned over periodically during ripening. The cheeses had a shape of eight lobes, a diameter of 27 cm, a height of 15 cm, and a weight 4–5 kg, depending on the ripening stage.

### 2.2. Sampling

Three independent replicate trials of cheeses were produced within an interval of 15 days at each dairy plant (D1 and D2), and eight cheeses were manufactured for each batch, for a total of 48 cheeses. For the chemical analysis, one cheese from each batch was taken randomly at 1, 15, 30, 60, 90, 120, 180, and 240 days of ripening. Cheeses were transported to the laboratory under refrigerated conditions (below 4 °C) and two sampling zones were studied, the external zone (Ext) and the internal zone (Int). A vertical cylinder section (6 cm diameter) cut from the cheese center was considered the internal zone (Int). After removing the rind (1 cm thick), eight cylinders of 3 cm in diameter (one per lobe) were cut perpendicular to the flat surface to sample the external zone (Ext). Cheese samples were analyzed the same day they were received for pH, dry matter content, and water activity. Aliquots of each sample were vacuum-packed and kept at −20 °C until the rest of the analytical determinations. A total of 96 samples (2 dairy plants × 3 batches × 8 ripening times × 2 sampling zones) were analyzed and each assay was carried out in duplicate.

### 2.3. Physicochemical Analysis

Water activity (a_W_) was measured directly using an Aqualab CX-2 dew-point hygrometer (Decagon Devices Inc., Pullman, USA). For pH measurement, ground cheese (10 g) was macerated with 50 mL of distilled water, and the pH of the resultant slurry was measured using an electrode Ag/AgCl (Crison pH 52–21) at 20 °C. Dry matter (DM) was analyzed according to the standards of the International Dairy Federation (ISO 5534:2004/IDF 4:2004) [11]. Total fat content was determined by the gravimetric method (ISO1735:2004/IDF 005) [12] in a Soxtec Avanti 2055 (Foss Tecator, Höganäs, Sweden) and drying in an oven (Memmert, Schwabach, Germany). Total nitrogen (TN) was determined by the Kjeldahl method (ISO 8961-1) [13] using a Kjeltec 8400 Analyzer (Foss Analytical, Höganäs, Sweden). Total protein content was calculated as TN multiplied by 6.38. Salt content (NaCl) was determined using an automatic titrator 848 Titrino Plus (Metrohm, Herisau, Switzerland) according to ISO 5943:2006/IDF 088 [14]. The method is based on potentiometric titration of chloride ions with a solution of silver nitrate (0.1 mol/L) and using the conversion factor 58.4 to calculate the concentration of sodium chloride in g NaCl/100 g DM. All analyses were performed in duplicate.

### 2.4. Proteolysis Assessment

The nitrogen fraction water soluble nitrogen (WSN), pH 4.4 soluble nitrogen (pH 4.4-SN), trichloroacetic acid 12% (*w*/*v*) soluble nitrogen (TCA-SN), and phosphotungstic acid soluble nitrogen (PTA-SN) were determined by International Dairy Federation FIL-IDF 25:1964 according to Butikofer et al. [15]. Four proteolytic indices were calculated as ratios between the various soluble fractions related to total nitrogen (TN): WSN/TN and pH 4.4-SN/TN as the ripening extension index, TCA-SN/TN as the ripening depth index, and PTA-SN/TN as the free amino acid index.

Free amino acids (FAAs) were determined with AccQ.Fluor Reagent^®^ from Waters. The free amino acid extraction was performed according to Milesi et al. [16] with some modifications. Cheese samples (5 g) were homogenized with 15 mL of distilled water and norleucine (500 µL, 25 mM) as internal standard using a Multi Reax shaker (Heidolph, Schwabach, Germany) for 15 min at 2000 rpm. The suspension was incubated in a water bath at 40 °C for 1 h and centrifuged at 4500 rpm for 30 min at 4 °C. The supernatant was filtered through fast-flow filter paper and the volume was made up to 25 mL with HCl 0.1 N. Afterward, the extract was filtered through 0.45 µm Millex membranes (Millipore, Molsheim, France) and diluted with HCl 0.1 N (1:4). Ten microliters of the diluted solution were mixed with 70 µL of borate buffer 0.2 M (pH 8.8) and 20 µL of the derivatization solution (10 mM; 6-aminoquinolyl-N-hydroxysuccinimidyl carbamate, AQC) as described by the manufacturer [17]. The reaction mixture was kept at room temperature for 1 min and then incubated in an oven at 55 °C for 10 min. The derivatized FAAs were separated, identified, and quantified by a reverse-phase high-performance liquid chromatography (HPLC) system Agilent 1100 Series (Agilent Technologies, Waldbronn, Germany) equipped with a diode-array detector (DAD) and a fluorescence detector (FLD). Chromatographic separation was carried out using a precolumn connected to a Kinitex 2.6 µm C18 100A column (100 mm × 4.6 µm) from Phenomenex (Torrence, CA, USA) at a temperature of 37 °C. Detection was performed with a fluorescence detector using an excitation wavelength of 250 nm and an emission wavelength of 395 nm, except for tryptophan, which was detected by DAD set at 254 nm. The injection volume was 5 µL and the flow rate was 1.3 mL/min. Data acquisition and processing were done with a ChemStation for LC system from Agilent Technologies.

Using the AQC method proposed by Waters Corporation, good resolution was achieved for 17 amino acid standards. However, for a complex matrix such as cheese, more amino acids are present, so a modified AQC derivatization method was needed. In this study, 25 amino acids were evaluated following the method proposed by Estrada et al. [18]: aspartic acid (Asp), asparagine (Asn), serine (Ser), glutamic acid (Glu), glycine (Gly), histidine (Hys), glutamine (Gln), taurine (Tau), arginine (Arg), citrulline (Cit), threonine (Thr), alanine (Ala), proline (Pro), γ-aminobutyric acid (GABA), α-aminobutyric acid (AABA), cysteine (Cys), tyrosine (Tyr), valine (Val), methionine (Met), ornithine (Orn), lysine (Lys), isoleucine (Ile), leucine (Leu), phenylalanine (Phe), and tryptophan (Trp). With the elution gradient shown in Supplementary Table S1, all AQC derivatives were eluted in 24 min.

### 2.5. Lipolysis Assessment: Analysis of Free Fatty Acids

Free fatty acids (FFAs) were analyzed according to the method described by Chavarri et al. [19] based on de Jong and Badings’s method [20]. Ground cheese (1.0 g) was homogenized with 3.0 g anhydrous sodium sulfate (Na_2_SO_4_), 0.3 mL 2.5 M sulfuric acid (H_2_SO_4_), and 100 µL internal standard solution (1 mg/mL pentanoic, nonanoic, and heptadecanoic acids). Lipids were extracted three times with 3 mL diethyl ether-heptane (1:1, v/v) and the solution was clarified by centrifugation. Organic phases containing FFAs and triacylglycerols were combined. The lipid extract was fractionated and triacylglycerols were separated from the FFAs on Sep-Pak aminopropyl Vac cartridges (Waters Corporation, Milford, Massachussetts) using a solid phase extraction (SPE) device GX-271 ASPEC (Gilson, Middleton, WI, USA). The column was equilibrated with 10 mL of heptane. After loading the lipid extract, triacylglycerols were eluted with 10 mL of chloroform–propanol (2:1, v/v) and FFAs were eluted with 5 mL diethyl ether containing 2% formic acid. The extract was analyzed the same day of extraction by gas chromatography (GC) on a HP 6890 Series GC (Agilent Technologies, Waldbronn, Germany) equipped with a flame ionization detector (FID) and HP-Innowax column (30 m × 0.32 mm id × 0.25 µm film thickness). Chromatographic conditions were as follows: 10 min at 65 °C, then up to a final temperature of 240 °C at 10 °C/min. Helium flow was set at 1 mL/min and a split ratio of 1:5. The FFAs were quantified by the internal standard method. Calibration curves were prepared by combining increasing concentrations (from 10 to 800 mg/L) of a mixture of butyric (C4:0), caproic (C6:0), caprylic (C8:0), capric (C10:0), lauric (C12:0), myristic (C14:0), palmitic (C16:0), palmitoleic (C16:1), stearic (C18:0), oleic (C18:1), linoleic (C18:2), and linolenic (C18:3) fatty acids. Ratios between the different fractions were calculated: short-chain fatty acids (SCFAs; C4:0–C8:0), medium-chain fatty acids (MCFAs; C10:0–C14:0), and long-chain fatty acids (LCFAs; C16:0–C18:3).

### 2.6. Statistical Analysis

Statistical analysis was performed using SPSS 19.0 (IBM Corp., Armonk, NY, USA). Data were analyzed using the general linear model (GLM) procedure and analysis of variance (ANOVA) was performed to establish the presence or absence of significant differences in the chemical composition, proteolysis, and lipolysis considering dairy plant and sampling zone as main factors and ripening time (1, 15, 30, 60, 90, 120, 180, and 240 days) as a co-variable. A *p*-value < 0.05 was considered statistically significant. Principal component analysis (PCA) was applied to nitrogen fractions and FAA data to reduce the variables to a minimum number of factors. Factors were rotated using the Varimax method to interpret the results.

## 3. Results and Discussion

### 3.1. Chemical Composition

The average values of pH, water activity (a_W_), dry matter (DM), fat, and protein in external and internal zones of cheeses manufactured at the two dairy plants after 1, 15, 30, 60, 90, 120, 180, and 240 days of ripening are shown in Table 1. Significant differences were observed in DM (*p* < 0.001), pH (*p* < 0.001), fat (*p* < 0.01), and protein (*p* < 0.001) between the two dairy plants, while water activity was not affected. Regarding sampling zone, a_W_ and pH were significantly lower in the external part, whereas DM was significantly higher in the external as compared to the internal part. As expected, a_W_ decreased while DM increased significantly during cheese ripening. Percentages of fat and protein did not show differences due to ripening time or sampling zone.

The artisanal cheesemaking process is one of the main reasons for the variations observed between dairies. Some differences in milk management before renneting, curd cutting, curd temperature, and cheesemaking technology are believed to be potentially responsible for different physicochemical composition in cheeses made under similar conditions [21]. In addition, the brining conditions could lead to significant differences in dry matter content, corresponding to different moisture levels of the cheeses, as reported by Todaro et al. [22] for Italian raw sheep milk cheeses.

The pH values ranged from 5.32 to 5.82 and significant differences between dairy plants were found, with cheeses from D1 showing lower pH values than those from D2. Likewise, the external parts of the cheeses showed lower pH values than the internal parts. Few studies have reported pH values in different parts of cheese. Malacarne et al. [7] and Sihufe et al. [9] did not find differences between zones in Parmigiano-Reggiano cheeses for this parameter. However, in agreement with our data, Tosi et al. [8] found higher pH values in the internal parts of Parmigiano-Reggiano than in external parts. Regardless of the different results found in other studies, our results fall within the range of pH allowed by PDO regulations of the European Commission for Spanish sheep milk cheeses such as Idiazábal (pH 5.1–5.8), La Serena (pH 5.2–5.9), Manchego (pH 4.8–5.8), Zamorano (pH 5.1–5.8), and Torta del Casar (pH 5.2–5.9).

At the beginning of the ripening period, there were no differences in water activity between the external and internal parts of the cheeses (0.98 initial value). However, throughout ripening, the external zone presented significantly lower a_W_ values than the internal zone. Pellegrino et al. [23] found the same phenomenon in Grana Padano cheese. Water activity value decreased 0.07 units (from 0.98 to 0.91) during 240 days of ripening, in agreement with other Spanish sheep milk cheeses such as Manchego [24], Idiazábal [25], and Los Pedroches [26].

The most important differences due to sampling zone were observed in dry matter and salt content, as reported in other cheese varieties such as Reggianito Argentino [9]. When cheeses are salted by brine immersion, salt is taken up while moisture is simultaneously lost. Comparing external and internal zones, no differences were found in dry matter levels at the beginning of ripening (55.5% on average). However, the differences in DM were gradually noticeable during ripening due to the natural and progressive loss of moisture, until reaching a 5–6 percentage unit difference at the end of the 240 days. These results contrast with other studies in which differences in dry matter between the external and internal zones were evident from the first stage of ripening. Thus, in Parmigiano-Reggiano cheese, the difference between internal and external zones was around 2–4% [7], and in Reggianito Argentino cheese it was 8–10% [9,27]. These varieties of cheese remain in brine for about 25 days and 9 days, respectively. However, our samples were kept in brine for only 12–24 h. Other varieties, such as Port Salut Argentino cheese packaged with plastic film, also showed a constant difference (4%) in DM between sampling zones, which remained constant throughout ripening [10].

In Grana Padano cheese [23] the DM difference between the center and the periphery of the cheese was 6%, similar to the difference observed between the parts analyzed in our study at 240 days of ripening. Although fat and protein contents were different in cheeses depending on the dairy plant, no significant differences in these parameters were observed between sampling zones during ripening.

### 3.2. Salt Distribution

**Figure 2:**
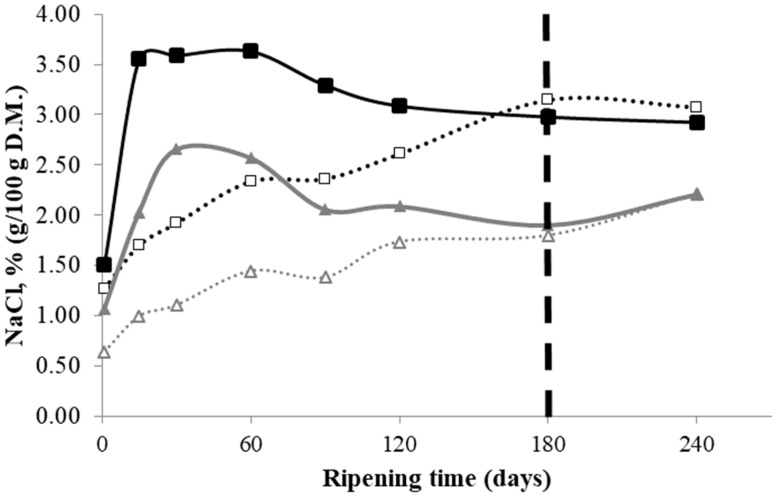
Sodium chloride content (g NaCl/100 g DM) in raw sheep milk cheeses made at two dairies (D1 and D2) during 240 days of ripening. (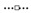): D1 internal zone, (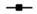): D1 external zone, (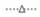): D2 internal zone, (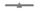): D2 external zone.

As expected, NaCl distribution changed during ripening. Figure 2 shows the NaCl content (g NaCl/100 g DM) in the cheese samples from both dairies in the internal and external zones during 240 days of ripening. At the beginning of maturation, the NaCl content in the outer zone (1.51% and 1.07% in D1 and D2, respectively) was significantly higher than in the inner zone (1.27% and 0.64% in D1 and D2, respectively) (*p* < 0.001). Regardless of the differences in final salt content at 240 ripening days (3.00% and 2.20% in D1 and D2, respectively), the phenomenon of salt diffusion from the outer zone into the cheese evolved similarly during ripening. Thus, homogeneous salt distribution was reached at 180 days of ripening in cheeses subjected to the two brining conditions. This suggests that the salt diffusion phenomenon occurred very consistently and was not adversely affected by the multilobed shape of the cheese.

Establishing the salt equilibrium in ripened cheese is a very slow process that can be affected by many factors, such as dairy plant, milk batch, brining conditions, and other cheesemaking processes [28]. The pH could have played a role in the final salt content of the cheeses, as higher pH values contribute to decreasing the negative charge of the casein micelles, leading to less retention of salt. In our case, the cheeses from the two dairies differed in their internal pH (5.38 in D1 versus 5.82 in D2 at 180 days of ripening), which corresponded to final salt contents of 3.0% and 2.2%, respectively. Additionally, cheeses with higher fat content generally show reduced rates of salt absorption, because the fat globules may obstruct the ducts where the brine enters the cheese. This phenomenon did not happen in our cheeses, since those with more fat content (55.6% in D1 versus 52.9% in D2) also reached a higher final salt concentration.

Although cheeses are manufactured under similar procedures, it is widely accepted that there are variations in the sensory attributes and compositional characteristics, mainly due to cheesemaking practices applied at each dairy [29]. In this study, cheeses salted in brine at 16°Baumé for 24 h had a 1% higher salt content than cheeses brined at 22°Baumé for 12 h.

### 3.3. Proteolysis 

#### 3.3.1. Nitrogen Fractions

**Figure 3:**
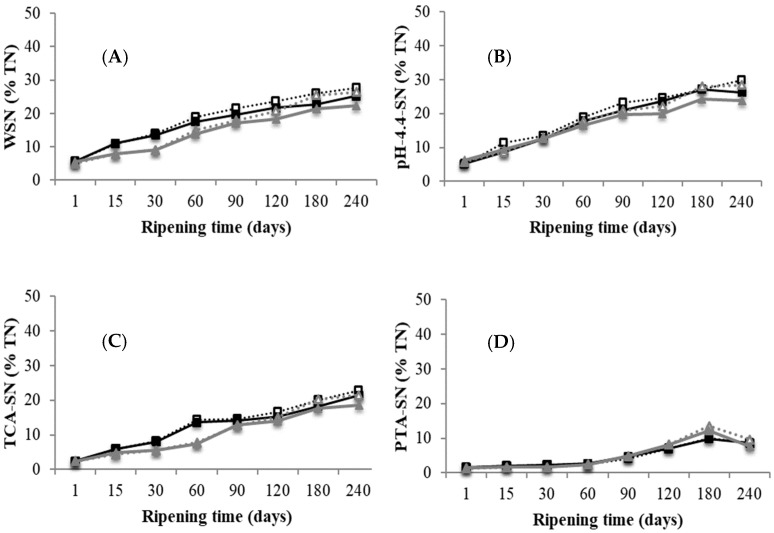
Soluble nitrogen fractions in raw sheep milk cheeses made at two dairies (D1 and D2) during 240 days of ripening (% of total nitrogen). (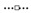): D1 internal zone, (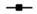): D1 external zone, (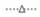): D2 internal zone, (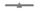): D2 external zone. (**A**) water-soluble nitrogen (WSN)/total nitrogen (TN); (**B**) pH 4.4 soluble nitrogen (SN)/TN; (**C**) trichloroacetic acid 12% (*w*/*v*) (TCA)-SN/TN; (**D**) phosphotungstic acid (PTA)-SN/TN.

The nitrogen fractions studied changed as a function of ripening time. Figure 3A,B reflect the extent of proteolysis (WSN/TN and pH 4.4-SN/TN), Figure 3C represents TCA-SN/TN as ripening depth index, and Figure 3D shows PTA-SN/TN as free amino acid index. The formation of soluble nitrogen compounds during cheese ripening is an index of the rate and extent of proteolysis, in that it is an indicator of casein hydrolysis brought about by the action of rennet and milk proteases present at the beginning of ripening. The extent of proteolysis during the studied period was moderate, as expected in raw sheep milk cheese made with calf rennet. WSN/TN reached a final value of around 25% total nitrogen.

These results are in agreement with those reported for similar varieties of raw sheep milk cheeses such as Manchego [24], Idiazábal [25], and Roncal [30]. The level of soluble nitrogen for each fraction increased significantly during the 240 days of ripening, while free amino acid content (PTA-SN/TN) decreased after 180 days of ripening. Several FAAs undergo transamination, decarboxylation, and deamination reactions rather extensively in late ripening [31]. This suggests that free amino acids enter other metabolic pathways from 180 days of ripening.

Significant differences (*p* < 0.05) between dairy plants were observed for the nitrogen fractions studied (WSN, pH 4.4-SN, TCA-SN, and PTA-SN). Cheeses made at D1 showed higher SN values for each nitrogen fraction than D2, while cheeses from D2 had lower salt content than those from D1. These results are in contrast to the inverse relationship between the extent of proteolysis and salt concentration widely reported in cheese [2,31]. Our results differed from those reported by other authors who studied other cheesemaking procedures with different amounts of rennet or dosages of starter cultures. Bustamante et al. [32] found that the amount of rennet had a significant effect (*p* < 0.01) on the extent of proteolysis (WSN/NT), and Galán et al. [33] concluded that the amount of rennet had more effect on proteolysis than the type of rennet used. Considering these results, the cheesemaking process has a greater influence on the degree of proteolysis than the salt concentration.

Regarding the sampling zone factor, no significant differences (*p* > 0.05) were found on the ripening index except for samples from dairy plant D2, which presented somewhat lower WSN/TN and pH 4.4-SN/TN at the external zone after 120 days of ripening. This agrees with the findings of Sihufe et al. [27], who reported that the degradation of casein was larger in the central zone than the external zone in Reggianito Argentino cheese, which may be related to the fact that the enzymatic system is favored by higher moisture content and lower salt content. Despite the differences found in salt concentration between the external and internal zones of the cheese until 180 days, the proteolysis index did not differ significantly between sampling zones.

#### 3.3.2. Free Amino Acids

The major individual amino acids were glutamic acid (Glu), lysine (Lys), asparagine (Asn), serine (Ser), ornithine (Orn), leucine (Leu), and phenylalanine (Phe), representing around 60% of the total free amino acids in the later stages of ripening (>90 days) (data not shown). Cysteine was not detected in any cheese samples and tryptophan was detected only in samples from D1 dairy.

Arginine concentration decreased later in ripening. This reduction is related to the metabolism of nonstarter lactic acid bacteria (NSLAB), which are able to convert arginine to citrulline and ornithine (not a constituent of casein) via the deiminase pathway [34]. This could be considered a positive aspect, as arginine is associated with bitter taste [35]. The effect of the sampling zone was not significant (*p* > 0.05) for most FAAs. Nevertheless, in the current samples, ornithine and arginine were inversely affected by sampling zone: while ornithine increased in the internal zone of cheeses during ripening, arginine concentration decreased. This could be associated with a higher growth of mesophilic lactobacilli in the internal zone due to lower salt content and higher water activity than in the external zone.

Gamma-aminobutyric acid (GABA), a nonprotein amino acid generated through decarboxylation of glutamic acid, has been correlated with an increased number of cheese eyes, although it has no direct or indirect impact on cheese flavor [36]. In this study, GABA values were around 3% of the total FAA at 240 days, which is similar to other raw sheep milk cheeses such as Idiazábal [37] and Manchego [38]. A higher concentration of GABA found in the internal zone could be related to the action of mesophilic lactobacilli [35]. Tyrosine was affected by sampling zone and its concentration was lower in the internal zone. In addition, its relative concentration decreased throughout ripening.

Total FAAs increased significantly during ripening, coinciding with concomitant increases in the concentrations of most individual FAAs, except for arginine and taurine. Although the sampling zone effect was not significant (*p* > 0.05) for most individual FAAs, significant differences were found for Arg, Tyr, and Orn at both dairies, and for GABA at D1. The Pearson correlation coefficient was calculated to estimate the relationship between individual amino acid concentrations and ripening time (Supplementary Table S2).

**Figure 4:**
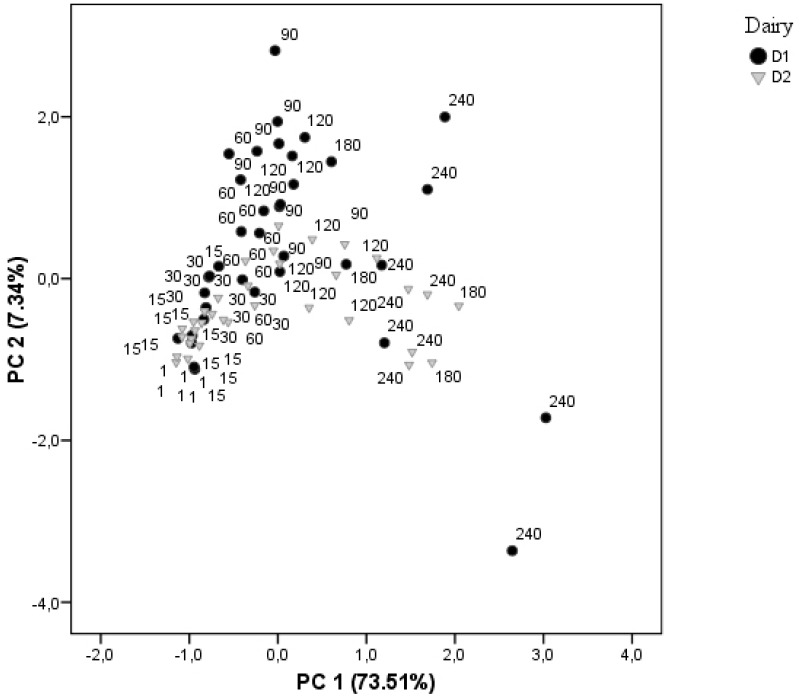
Plot of first two principal components obtained from principal component analysis of proteolysis data of cheeses from two dairies (D1 and D2) during ripening (1, 15, 30, 60, 90, 120, 180, and 240 days).

Principal component analysis (PCA) of proteolysis data (nitrogen fractions, individual and total FAA concentrations) distributed the samples according to ripening time and dairy plant. Two principal components, PC1 (73.51%) and PC2 (7.34%), explained 80.85% of the variance (Figure 4). The score plot organizes samples with long ripening (>60 days) according to dairy plant. However, younger cheeses (<60 days) were grouped together. PC1 presented high correlation with most individual amino acids (apart from Tau, Arg, and Tyr), while PC2 showed the highest correlations with SN-pH4.4/NT, WSN/NT, and leucine content.

### 3.4. Lipolysis

**Figure 5:**
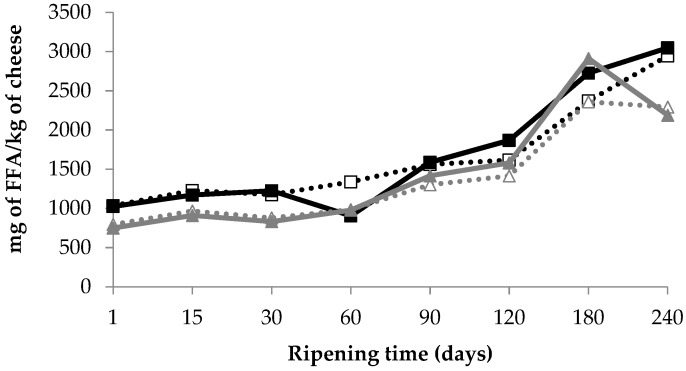
Total free fatty acid (FFA) values expressed as sum of individual FFAs in raw sheep milk cheeses made at two dairies (D1 and D2) during 240 days of ripening. (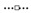): D1 internal zone, (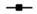): D1 external zone, (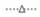): D2 internal zone, (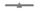): D2 external zone.

The extent of lipolysis was moderate as expected in semi-hard/hard cheese varieties due to low lipolytic activity of lactic acid bacteria [39]. Total FFA values expressed as the sum of individual FFAs during ripening are shown in Figure 5. Total FFA levels increased significantly (*p* < 0.05) during ripening in samples from both dairies and were significantly higher (*p* < 0.01) in samples from D1 (~3000 mg FFA/kg of cheese) than from D2 (~2250 mg FFA/kg of cheese) at the end of ripening. However, no significant variation in the composition of total FFA was observed in the two sampling zones. Differences in FFA concentration may be significant even in the same cheese variety. The intra-variety difference in total FFA has been related to milk composition and microbial activity, depending on sheep breed, stage of lactation, season, and feeding, and many studies on cheeses manufactured at different dairy plants corroborate this variability [40].

The comparison between sampling zones showed a uniform distribution of FFAs for Reggianito Argentino at 4 months of ripening, in agreement with the data reported by Sihufe et al. [9]. However, Malacarne et al. [7] found higher FFA content in the external zone of Parmigiano-Reggiano cheese at 24 months of ripening. Samples with higher salt content (D1) showed a greater extent of lipolysis. Previous studies in Cheddar cheese manufactured with pasteurized milk indicated that NaCl may inhibit lipolysis [41]. However, other investigations in Cheddar cheese concluded that FFAs were unaffected by salt reduction [6]. This suggests that the FFA values in our study could be ascribed to different milk components such as lipoprotein lipase (LPL), the principal indigenous lipase in raw milk, as well as to microbial growth [39].

The individual FFAs were grouped into short-chain fatty acids (C4:0–C8:0, SCFAs), medium-chain fatty acids (C10:0–C14:0, MCFAs), and long-chain fatty acids (C16:0–C18:3, LCFAs), and were expressed as percentages of total FFA. The initial proportion of LCFA (65% of total FFA) decreased during ripening to 45%. LCFAs have a higher perception threshold and do not contribute much to cheese flavor. Relative concentration of MCFA (25–30%) did not show significant variation during ripening. However, SCFA proportion increased during the study period from 10 to 25%. It is usually considered that short-chain fatty acids have a significant impact on the development of the characteristic cheese flavor [39]. Our results show a preference of lipases for short-chain fatty acid residues, since these acids predominate at the sn-3 position. This has been reported in other cheeses such as Idiazábal [19], Manchego [24], and Queso Picante [42].

As a result of lipolysis, all studied individual FFA concentrations increased significantly (*p* < 0.001) with ripening time. The major FFAs were palmitic (C16:0) and oleic (C18:1) acids, coinciding with other sheep milk cheeses [43]. Pearson correlation coefficients between the percentage of FFA and ripening time were calculated (Supplementary Table S3). The proportions of butyric (C4:0) and caproic (C6:0) acids, which are known to contribute actively to cheese flavor and aroma, showed the highest correlation coefficient (*r* > 0.80). Despite the statistical differences found for some FFAs among dairy plants, all samples showed a similar pattern of lipolysis.

## 4. Conclusions

In this artisanal hard cheese, salt was homogeneously distributed in the entire product at 180 days of ripening. The multilobed shape of the cheese did not adversely affect the rate of salt absorption. The brining conditions (time and salinity) had no influence on the rate of salt penetration. However, they had a significant effect on NaCl content, as cheeses salted in brine solution at 16°Baumé for 24 h showed a higher salt content than cheeses brined at 22°Baumé for 12 h. Proteolysis and lipolysis showed similar patterns in all samples and evolved homogeneously during the ripening period, in both the internal and external zones of cheese. These findings may constitute useful information for cheese producers in deciding when to market this product in order to reach uniformity in salt distribution.

## Figures and Tables

**Table 1:** Mean values ± SD of chemical variables in raw sheep milk cheeses from two dairies (D1 and D2) sampled at external and internal zones during ripening.

Dairy Plant	Days of Ripening	pH		Water Activity (a_W_)		Dry Matter (DM)		Fat (% DM)		Protein (% DM)		NaCl (% DM)	
		Internal	External	Internal	External	Internal	External	Internal	External	Internal	External	Internal	External
D1	1	5.46 ± 0.15	5.45 ± 0.16	0.98 ± 0.00	0.98 ± 0.01	54.3 ± 1.0	54.1 ± 1.6	54.5 ± 2.5	52.4 ± 1.9	37.7 ± 1.1	37.2 ± 0.6	1.27 ± 0.08	1.51 ± 0.29
	15	5.47 ± 0.09	5.46 ± 0.04	0.98 ± 0.00	0.97 ± 0.00	55.9 ± 5.1	54.6 ± 2.3	50.9 ± 3.3	51.6 ± 1.6	35.5 ± 3.9	35.5 ± 2.5	1.70 ± 0.13	3.55 ± 0.17
	30	5.48 ± 0.09	5.53 ± 0.08	0.97 ± 0.01	0.96 ± 0.01	55.1 ± 1.8	55.8 ± 0.4	52.8 ± 3.8	50.6 ± 3.5	37.0 ± 1.1	36.1 ± 1.5	1.92 ± 0.43	3.59 ± 0.33
	60	5.47 ± 0.12	5.52 ± 0.05	0.97 ± 0.00	0.95 ± 0.00	57.0 ± 0.7	58.9 ± 1.2	53.6 ± 2.2	51.7 ± 2.2	37.1 ± 0.1	36.0 ± 1.0	2.34 ± 0.32	3.63 ± 0.12
	90	5.45 ± 0.05	5.37 ± 0.05	0.96 ± 0.00	0.95 ± 0.00	59.3 ± 0.6	61.3 ± 1.7	52.3 ± 3.8	53.5 ± 5.9	37.2 ± 0.8	37.4 ± 1.6	2.36 ± 0.05	3.29 ± 0.07
	120	5.46 ± 0.11	5.41 ± 0.09	0.96 ± 0.00	0.95 ± 0.00	60.0 ± 1.1	62.8 ± 2.0	55.0 ± 5.6	53.0 ± 2.5	38.8 ± 3.3	36.3 ± 0.6	2.61 ± 0.32	3.09 ± 0.29
	180	5.38 ± 0.13	5.32 ± 0.16	0.94 ± 0.00	0.93 ± 0.01	63.6 ± 1.3	69.1 ± 2.9	55.6 ± 2.3	48.3 ± 3.5	36.3 ± 0.9	36.4 ± 0.8	3.15 ± 0.13	2.98 ± 0.26
	240	5.54 ± 0.11	5.43 ± 0.07	0.92 ± 0.00	0.91 ± 0.00	66.6 ± 1.1	71.8 ± 0.4	52.5 ± 2.6	52.8 ± 1.7	36.5 ± 0.6	36.6 ± 0.6	3.07 ± 0.26	2.92 ± 0.28
D2	1	5.63 ± 0.03	5.58 ± 0.03	0.98 ± 0.00	0.98 ± 0.01	57.5 ± 1.4	56.1 ± 1.5	51.8 ± 2.8	51.2 ± 2.4	40.1 ± 1.4	40.6 ± 1.0	0.64 ± 0.34	1.07 ± 0.51
	15	5.71 ± 0.11	5.77 ± 0.12	0.97 ± 0.01	0.98 ± 0.00	58.2 ± 2.0	58.1 ± 1.3	49.9 ± 2.3	50.0 ± 2.6	39.9 ± 2.6	39.1 ± 2.3	0.99 ± 0.44	2.03 ± 0.97
	30	5.73 ± 0.04	5.65 ± 0.07	0.97 ± 0.00	0.96 ± 0.01	59.2 ± 1.0	58.7 ± 1.1	50.7 ± 3.0	50.6 ± 2.2	39.7 ± 1.9	39.2 ± 1.8	1.1 ± 0.38	2.66 ± 0.54
	60	5.61 ± 0.08	5.58 ± 0.15	0.97 ± 0.00	0.95 ± 0.01	60.8 ± 1.3	63.2 ± 1.4	51.3 ± 2.6	50.3 ± 2.8	37.5 ± 3.8	39.5 ± 1.4	1.44 ± 0.50	2.57 ± 0.55
	90	5.75 ± 0.04	5.60 ± 0.01	0.96 ± 0.00	0.95 ± 0.01	62.8 ± 0.7	66.3 ± 2.0	51.0 ± 1.3	50.5 ± 2.5	39.9 ± 0.8	39.3 ± 1.3	1.38 ± 0.36	2.06 ± 0.27
	120	5.81 ± 0.18	5.61 ± 0.27	0.95 ± 0.01	0.94 ± 0.01	64.3 ± 0.9	69.0 ± 1.1	52.1 ± 3.9	49.1 ± 2.8	40.5 ± 0.6	38.5 ± 3.4	1.73 ± 0.59	2.09 ± 0.51
	180	5.82 ± 0.17	5.53 ± 0.13	0.94 ± 0.00	0.93 ± 0.01	67.2 ± 0.3	72.3 ± 0.6	52.9 ± 4.0	49.3 ± 2.0	38.7 ± 0.8	38.4 ± 0.5	1.8 ± 0.61	1.9 ± 0.59
	240	5.76 ± 0.03	5.59 ± 0.13	0.93 ± 0.00	0.91 ± 0.01	68.1 ± 0.2	74.4 ± 1.0	48.8 ± 0.2	48.4 ± 0.0	40.6 ± 3.8	38.6 ± 1.7	2.21 ± 0.22	2.21 ± 0.57
Dairy plant (D)		***		NS		***		**		***		***	
Sampling zone (SZ)		**		***		***		NS		NS		***	
Ripening time		NS		***		***		NS		NS		*	

Significance levels: ***, *p* < 0.001; **, *p* < 0.01; * *p* < 0.05; NS, not significant; SD, standard deviation; NaCl, sodium chloride.

## Supplementary Materials

The following are available online at https://www.mdpi.com/2304-8158/8/3/100/s1: Table S1. Quaternary gradient for separation of free amino acids in cheese derivatized with 6-aminoquinolyl-N-hydroxysuccinimidyl carbamate (AQC) by high-performance liquid chromatography (HPLC-DAD-FLD). Table S2. Significance of ripening time, dairy plant, and sampling zone on individual concentration of free amino acids (FFAs) and total FAA, and Pearson coefficient (r) in raw sheep milk cheeses made in two dairies (D1 and D2) during 240 days of ripening. Table S3. Significance of ripening time, dairy plant, and sampling zone on relative concentrations of individual free fatty acids (FFAs) and Pearson coefficient (r) in raw sheep milk cheeses made in two dairies (D1 and D2) during 240 days of ripening.
