# Supplementary material for: Salt Distribution in Raw Sheep Milk Cheese during Ripening and the Effect on Proteolysis and Lipolysis
Source: Foods. 2019 Mar 17;8(3):100. doi: 10.3390/foods8030100 (PMC6462923; doi:10.3390/foods8030100)
Supplement: Supplementary file 1 [file foods-08-00100-s001.pdf]

Supplementary Table S1. Quaternary gradient for the separation of free amino acids in cheese derivatized with AQC by high performance liquid chromatography (HPLC-DAD-FLD).

Gradient	Mobile phases			
Time (min)	% A* (pH: 4.90)	% B (acetonitrile)	% C (water)	%D* (pH: 5.15)
0.00	100	0	0	0
0.40	98.7	1.3	0	0
3.84	97.5	2.5	0	0
4.00	97.5	2.5	0	0
7.76	96.3	3.7	0	0
11.28	95.1	4.9	0	0
14.80	94	6	0	0
15.60	92	8	0	0
16.40	91	9	0	0
17.20	90	10	0	0
20.50	0	21	0	79
23.00	0	22	0	78
27.00	0	60	40	0
30.00	100	0	0	0

*: AccQ.Tag eluent solution prepared according to the manufacturer specification (Waters)

Supplementary Table S2. Significance of the effects 'Ripening time', 'Dairy plant' and 'Sampling zone' on the individual concentration of free amino acids (FFA) and total FAA, and Pearson coefficient (r) in raw sheep milk cheeses made in two dairies (D1 and D2) during 240 days of ripening.

	Dairy plant	Dairy plant D1			Dairy plant D2		
		(*r*)	Ripening time	Sampling zone	(*r*)	Ripening time	Sampling zone
Asp	*	0.770	***	NS	0.924	***	NS
Asn + Ser	***	0.817	***	NS	0.950	***	NS
Glu	**	0.879	***	NS	0.962	***	NS
Gly	NS	0.853	***	NS	0.913	***	NS
Hys + Gln	***	0.853	***	NS	0.913	***	NS
Tau	NS	0.155	NS	NS	-0.105	NS	NS
Arg	NS	0.080	NS	*	0.162	NS	*
Cit + Thr	*	0.688	***	NS	0.948	***	NS
Ala	**	0.876	***	NS	0.928	***	NS
Pro	*	0.804	***	NS	0.929	***	NS
Gaba	*	0.677	***	***	0.595	***	NS
Cys	ND	ND	-	-	ND	-	-
Tyr	*	0.421	**	*	0.684	***	**
Val	NS	0.812	***	NS	0.851	***	NS
Met	***	0.866	***	NS	0.970	***	NS
Orn	**	0.905	***	**	0.903	***	*
Lys	***	0.939	***	NS	0.935	***	NS
Ile	*	0.937	***	NS	0.925	***	NS
Leu	NS	0.840	***	NS	0.819	***	NS
Phe	NS	0.877	***	NS	0.899	***	NS
Trp	NS	0.573	**	NS	ND	-	-
Total FAA	**	0.924	***	NS	0.947	***	NS

Significance levels: ***, P < 0.001; **, P < 0.01; *P < 0.05; NS, not significant; ND: not detected.

Supplementary Table S3: Significance of the effects 'Ripening time', 'Dairy plant' and 'Sampling zone' on relative concentrations of individual free fatty acids (FFA) and Pearson coefficient (r) in raw sheep milk cheeses made in two dairies (D1 an D2) during 240 days of ripening.

	Ripening time	Dairy plant	Sampling zone	Dairy plant D1 (*r*)	Dairy plant D2 (*r*)
% C4:0	***	***	NS	0.87	0.85
% C6:0	***	***	NS	0.90	0.80
% C8:0	***	NS	NS	0.79	0.56
% C10:0	NS	***	NS	0.15	0.19
% C12:0	NS	**	NS	0.02	-0.01
% C14:0	**	***	NS	0.54	0.21
% C16:0	***	**	NS	-0.28	-0.53
% C16:1	**	NS	NS	-0.28	-0.34
% C18:0	***	*	NS	-0.64	-0.70
% C18:1	**	NS	NS	-0.50	-0.17
% C18:2	***	NS	NS	-0.53	-0.62
% C18:3	***	NS	NS	-0.83	-0.86

Significance levels: ***, P < 0.001; **, P < 0.01; *P < 0.05; NS, not significant.
